# Impact of COVID-19 on the Gastrointestinal Tract: A Clinical Review

**DOI:** 10.7759/cureus.23333

**Published:** 2022-03-20

**Authors:** Haider Ghazanfar, Sameer Kandhi, Dongmin Shin, Aruna Muthumanickam, Hitesh Gurjar, Zaheer A Qureshi, Mohammed Shaban, Mohamed Farag, Asim Haider, Pravash Budhathoki, Tanushree Bhatt, Ali Ghazanfar, Abhilasha Jyala, Harish Patel

**Affiliations:** 1 Internal Medicine, BronxCare Health System, Bronx, USA; 2 Internal Medicine, Icahn School of Medicine at Mount Sinai, New York, USA; 3 Internal Medicine, University of Health Sciences, Lahore, PAK; 4 Internal Medicine, Bronxcare Health System, Bronx, USA; 5 Cardiology, Federal Medical and Dental College, Islamabad, PAK; 6 Medicine/Gastroenterology, BronxCare Health System, Bronx, USA

**Keywords:** pancreas, liver, angiotensin converting enzyme 2, gastrointestinal tract, covid-19

## Abstract

Coronavirus disease 2019 (COVID-19) has spread rapidly throughout the world, causing a pandemic that has resulted in more than 5 million deaths globally. The gastrointestinal (GI) tract is known to have high expression of angiotensin-converting enzyme 2 (ACE2) receptors in the human body, making it prone to direct damage from the cellular invasion of severe acute respiratory syndrome coronavirus 2 (SARS-CoV-2). Numerous GI symptoms have been reported among patients with COVID-19. This systemic review details the mechanism and effects of COVID-19 on the GI tract along with the hepatobiliary and pancreatic systems.

## Introduction and background

Coronavirus disease 2019 (COVID-19), caused by the highly contagious severe acute respiratory syndrome coronavirus 2 (SARS-CoV-2), was first reported in December 2019 in Wuhan, China [1]. It had quickly spread worldwide, leading to a pandemic that devastated our society for the past two years. As of January 7, 2022, over 299 million cases have been reported worldwide with over 5 million deaths [2].

The major pathogenesis of COVID-19 involves the attachment of the SARS-CoV-2 spike protein to the angiotensin-converting enzyme 2 (ACE2) receptors expressed on the host cell surface, leading to viral entry into the cell. ACE2 receptors are expressed throughout various human tissues with the highest expression found in the alveolar cells of the lung and the epithelial cells of the gastrointestinal (GI) tract, which serve as possible routes of infection [3].

Numerous GI symptoms have been reported in the literature among patients with COVID-19, with the incidence of these symptoms ranging widely from 2% to 79.1% [4-6]. Certain GI illnesses also pose a risk for severe COVID-19 infection. The widespread use of glucocorticoids in inflammatory GI disorders and the everyday use of proton pump inhibitors (PPIs) have both been linked to severe clinical outcomes in COVID-19 infection [7-8].

## Review

Clinical and pathologic evidence for COVID-19 involving the digestive system

**Figure 1:**
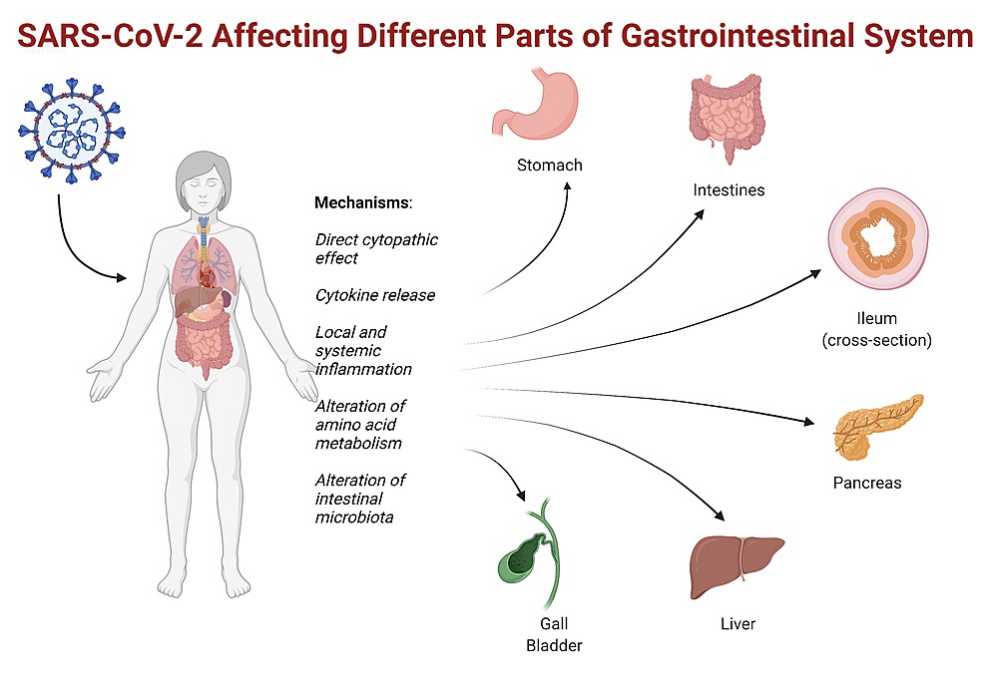
Effect of SARS-CoV-2 on the GI organ system SARS-CoV-2: severe acute respiratory syndrome coronavirus 2; GI: gastrointestinal

Since the beginning of the pandemic, the clinical presentation of symptomatic COVID-19 has been predominantly respiratory and systemic symptoms. However, ongoing studies on the disease have shown significant clinical and pathologic evidence of COVID-19 involving the digestive system as well. The effect of SAR-CoV-2 on the GI organ system is shown in Figure 1.

A meta-analysis of 60 studies that included 4243 COVID-19 patients showed a pooled prevalence of 17.6% for all GI symptoms. Symptoms included anorexia (26.8%), diarrhea (12.5%), nausea/vomiting (10.2%), and abdominal pain/discomfort (9.2%) [6]. Another recent meta-analysis done by Borges et al., which involved 59254 patients from 11 countries, showed that 9% of the patients displayed gastrointestinal symptoms [9]. Parasa et al. analyzed 23 published and six preprint studies in 2020 and found that out of the total of 4805 patients, 7.4% had diarrhea, and 4.6% had nausea or vomiting [10].

Importantly, there have been few reports of isolated infectious SARS-CoV-2 from stool, which likely confirmed the release of the infectious virions to the gastrointestinal tract, raising the possibility of fecal-oral transmission as an additional route for the virus to spread [11-12]. Though not fully established as a mode of transmission, the fecal transmission of SARS-CoV-2 has been shown in animal studies [13].

In one study, viral ribonucleic acid (RNA) was detected in stool in 40.5% of patients and SARS-CoV-2 remained positive in the stool even after the virus has cleared up from the respiratory tract [10]. Zuo et al. isolated fecal SARS-CoV-2 RNA in 47% of patients with COVID-19 in the absence of GI symptoms [14]. In another study out of the 42 COVID-19 patients, 18 (64.29%) were found to have virus RNA in the feces for about seven days after negative conversion of the pharyngeal swabs [15].

Shedding of SARS CoV-2 RNA from the GI tract was variable with the course of infection. Zheng et al. found that the positive rate in stool samples gradually increased from the first week and then decreased from the third week [16]. The viral load of stool samples was highest during the third and fourth weeks after disease onset. The duration of SARS-CoV-2 positivity is significantly longer in stool samples than in respiratory and serum samples [16]. A cohort study on 74 confirmed COVID-19 patients showed that viral shedding in feces can be extended as long as five weeks after clearing viral RNA from respiratory samples. The average duration of positive respiratory samples was 16.7 days while fecal samples were positive for a mean of 27.9 days. Such findings raise attention to the possible need for an extended period of isolation in select cases and adding the fecal SARS-CoV-2 test to aid the decision on when to discontinue precautions to prevent the transmission of the disease [17]. Zhang et al. reported nucleic detection of COVID-19 in stool specimens to be equally accurate compared to pharyngeal swab specimens. They also observed that a positive stool test did not correlate with GI symptoms or with the severity of the lung infection [18].

Adding further support of pathologic evidence, Xiao et al. found proof of GI infection of SARS-CoV-2 through RNA detection and intracellular staining of the ACE2 receptor and viral nucleocapsid protein in gastric, duodenal, and rectal epithelia [11]. Similarly, in 2003, Leung et al. reported that coronaviruses could cause active viral replication in the intestinal tract of infected patients, evidenced by electronic microscopy and viral culture [19].

GI conditions increasing the risk of COVID-19

Several GI diseases have been implicated in increasing the risk of COVID-19.

Inflammatory Bowel Disease (IBD)

With the outbreak of COVID-19, there have been concerns regarding whether patients with IBD should continue their immunomodulators and biologic agents. There has been growing consensus on continuing immunosuppressive treatment along with minimizing the risk of infection through basic protective measures such as frequent hand washing, shielding, and social distancing. It is believed that medication discontinuation would lead to an increased risk of relapses, hospitalizations, and the need for more aggressive therapeutic interventions [20]. Patients at the highest risk are thought to be those with active IBD disease, those in need of induction therapy with steroids or biological agents, elderly patients, and those with comorbidities. Patients under monotherapy or combination therapy were advised to strictly implement extended social distancing [6]. In 2020, the International Organization for the Study of Inflammatory Bowel Disease (IOIBD) issued detailed guidance about IBD and COVID-19. It is uncertain if active inflammation from IBD increases the risk of SARS-CoV-2 infection. It is recommended that 5-aminosalicylic acid therapy should be continued in patients with COVID-19 [21]. Budesonide therapy should not be discontinued, and its dose should not be decreased in order to prevent SARS-CoV-2 infection [21]. However, it is uncertain if budesonide or vedolizumab should be stopped in the setting of SARS-CoV-2 infection. Patients on anti-tumor necrosis factor (TNF) or ustekinumab should discontinue treatment if they develop COVID-19. In addition, patients on prednisone ≥ 20 g/day, 6-mercaptopurine (MP), azathioprine, methotrexate, and tofacitinib should discontinue treatment once they test positive for SARS-CoV-2. IBD medications can be restarted after the resolution of COVID-19 symptoms and after two nasopharyngeal polymerase chain reaction tests are negative [21]. The Surveillance Epidemiology of Coronavirus Under Research Exclusion for Inflammatory Bowel Disease (SECURE-IBD) registry has been created to monitor the outcomes of COVID-19 in IBD patients worldwide. Data gathered from the initial 525 cases in 33 countries revealed that the risk factors for severe COVID-19 among patients with IBD included increasing age, two or more comorbidities, systemic corticosteroids, and sulfasalazine or 5-aminosalicylate use. On the contrary, tumor necrosis factor antagonist treatment was not associated with poor outcomes [7]. An updated SECURE-IBD registry with 7,038 patients showed a similar pattern. The overall mortality rate is 2% among IBD patients, which is not significantly higher than the general population. Risk for adverse outcomes, such as intensive care unit (ICU) admission, ventilator use, or death, increases with increasing age and comorbidities. Systemic corticosteroids are associated with the highest risk (12%) for adverse outcomes among IBD medications, followed by methotrexate monotherapy (7%), 6-MP/azathioprine monotherapy (6%), sulfasalazine/mesalamine (6%), budesonide (6%). On the other hand, monotherapy with anti-TNF treatment or interleukin (IL) 12/23 inhibitor was associated with poor outcomes in only 1-2% of cases [6,22].

Chronic Liver Disease (CLD)

The presence of chronic liver disease is considered a risk factor for a worse prognosis in COVID-19 [23-24]. Outcome data in CLD patients with SARS-CoV-2 infection indicate a significantly increased risk of death. Patients with cirrhosis can also have an underlying hepatopulmonary syndrome, portopulmonary hypertension, or hepatic hydrothorax, which can all increase the risk of respiratory failure and contribute to the worse outcome. This risk increases with the severity of the liver disease, reflected by the Child-Turcotte-Pugh (CTP) class or model for end-stage liver disease (MELD) score. In addition, CLD is commonly associated with other comorbidities, such as obesity and metabolic syndrome, which has a negative impact on the prognosis of COVID-19. Hepatic decompensation was reported frequently amongst cirrhotic patients with concurrent COVID-19 infection, even without respiratory symptoms [25-29]. Williamson et al. analyzed the records of 10,926 patients who died of COVID-19 and concluded that liver disease was associated with an increased risk of death [30]. Early reports of COVID-19 revealed liver function test abnormalities correlating with disease severity. The most common laboratory abnormalities were increased serum aminotransferases. Hypoalbuminemia has also been linked with severe disease, even in patients without chronic illness [31]. Possible mechanisms of liver injury in COVID-19 include direct invasion of the virus causing injury, indirect immune-mediated damage from systemic inflammatory response, hypoxia, hemodynamic instability, and coagulopathy related to the disease leading to ischemic injury, iatrogenic causes such as drug-induced liver injury [32].

Gastroesophageal Reflux Disease (GERD) and Peptic Ulcer Disease (PUD)

PPIs are the most effective inhibitor of acid secretion in the stomach. PPIs are commonly used to treat gastroesophageal reflux disease and peptic ulcer disease. According to a study done in 2004, the highly acidic condition of the stomach of pH < 3 completely inactivated SARS-CoV [33]. Similarly, Zhou et al. demonstrated that viruses pseudotyped with the SARS-CoV-2 spike protein were completely inactivated under the pH of 1.0 and 2.0, a condition similar to the normal acidity of a stomach. Chronic PPI use would decrease gastric acidity and subsequently increase the chance of SARS-CoV-2 entering the gut, which has a high expression of ACE2 receptors [34].

One study that analyzed 3386 COVID-19 cases found evidence of an independent, dose-response relationship between the use of PPI and SARS-CoV-2 positivity. Those taking PPIs twice a day had higher odds for reporting a positive test compared with individuals on lower-dose PPIs. Individuals taking less-potent histamine-2 receptor antagonists were not at increased risk [35]. A more comprehensive analysis including 14,163 current PPI users and 6242 past PPI users found that among those with COVID-19, the current use of PPIs was associated with a 79% higher risk for worse outcomes, and up to 90% increased risk of severe outcomes if PPI use was started within the previous 30 days. However, the SARS-CoV-2 positivity rate was not associated with current or past use of PPIs [8]. These findings lead to suspicion for possible confounding by indication since patients with more severe disease may require PPI due to high-dose corticosteroid use and for stress ulcer prophylaxis for critically ill patients at high risk for GI bleeding such as those on mechanical ventilation [36].

GI Malignancy

Cancer patients are estimated to have a two-fold increased risk of SARS-CoV-2 infection compared to the general population, as they are likely to be immunocompromised secondary to their underlying malignancy and chemotherapy [37].

Mechanisms of damage to the GI tract during COVID-19 infection

Potential mechanisms on how SARS-CoV-2 can cause damage to the GI tract include direct virus-induced cytopathic effect through cell entry via ACE2, indirect immune-mediated injury triggered by a systemic inflammatory response to SARS-CoV-2, disruption of the intestinal microecological balance leading to excessive inflammation of the gut and lung, which may lead to a cytokine storm through the gut-lung axis and drug-related injuries.

Interaction Between the SARS-CoV-2 Spike Protein and ACE2 Receptors

**Figure 2:**
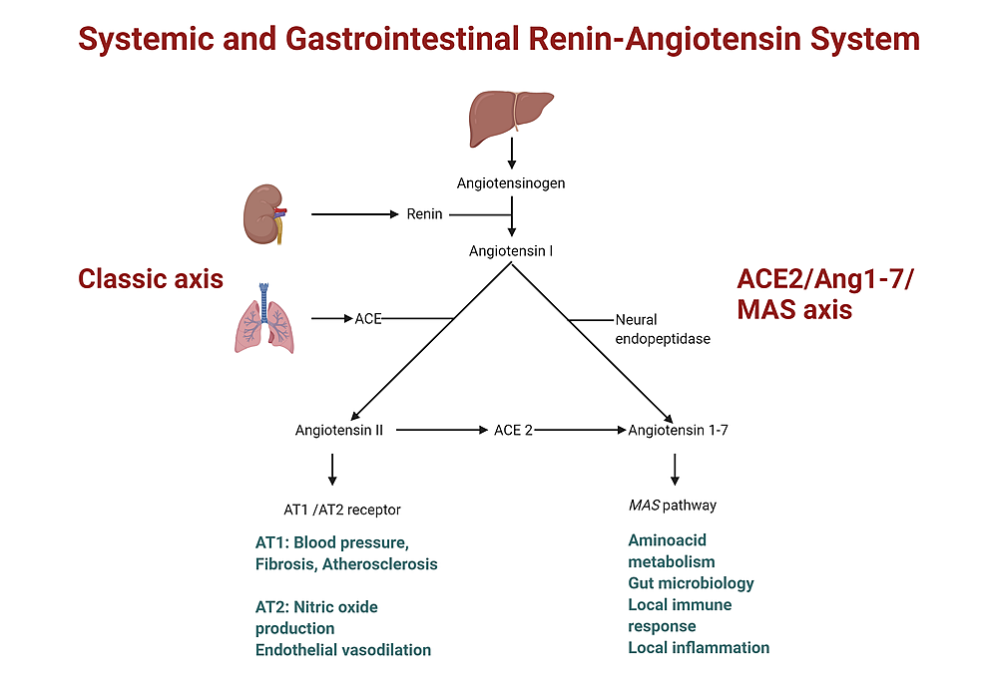
Illustrated diagram of the systemic and GI renin-angiotensin system ACE 2: angiotensin-converting enzyme 2; Ang1-7: angiotensin-(1–7); AT1: angiotensin II type 1 receptor; AT2: angiotensin II type 2 receptor

It has been proven that the entry of SARS-CoV-2 into epithelial cells is mediated by the binding of the viral spike (S) proteins to the ACE2 receptor expressed on cells. The S protein has S1 and S2 functional subunits. S1 facilitates viral attachment to the surface of target cells and S2 allows fusion of the viral and cellular membranes. Additionally, viral entry into target cells also requires S protein priming by cellular transmembrane serine protease 2 (TMPRSS-2), which cleaves the S protein at the S1/S2 and S2’ sites. SARS CoV-2 infection can cause severe host hyperimmune response with a life-threatening cytokine storm that may result in systemic inflammatory response syndrome [38]. The S protein of SARS-CoV-2 has a 10-20 times greater affinity to ACE2 compared to that of SARS-CoV, which may explain the higher transmission rate of SARS-CoV-2 [39]. The systemic and GI renin-angiotensin system has been presented in Figure 2.

A GI infection of SARS-CoV-2 has been evidenced by the isolation of viral RNA from GI epithelial cells and positive intracellular staining of a viral nucleocapsid protein in gastric, duodenal, and rectal glandular epithelial cells, as mentioned previously. Positive staining of ACE 2 receptors in the cytoplasm of GI epithelial cells shows that ACE2 receptors are widely expressed through the GI tract. However, ACE2 staining was rarely seen in the esophageal epithelium, which is mainly composed of squamous epithelial cells that express less ACE2 than glandular epithelial cells. This is in accordance with the absence of viral nucleocapsid protein staining in the esophageal mucosa. Zang et al. found that the expression of ACE2 is significantly higher in small intestines than in all other organs, including the lungs. They also suggested that SARS-CoV-2 infects human small intestinal enterocytes and actively replicates in ACE2-expressing mature enterocytes. Mucosa-specific serine proteases, transmembrane protease serine 2 (TMPRSS2), and transmembrane protease serine 4 (TMPRSS4) facilitated SARS-CoV-2 spike fusogenic activity and promoted viral entry into enteroid cells.

In COVID-19 patients, ACE2 expression is downregulated due to the internalization of the ACE2 attached to the virus. Subsequent accumulation of angiotensin II level is directly related to the severity of the disease and lung injury [40]. However, in the intestine, instead of functioning primarily through the renin-angiotensin system (RAS), ACE2 serves as an important regulator of intestinal amino acid homeostasis, expression of antimicrobial peptides, gut microbial ecology, and intestinal inflammation [41]. Ye et al. suggested that binding of SARS-CoV-2 with ACE2 in the gastrointestinal tract reduces the level of available receptors, affecting tryptophan absorption that may disrupt the steady state of the intestinal flora, leading to gastrointestinal symptoms such as diarrhea [42].

Apart from ACE2, other receptors also play a role in SARS-CoV-2 entry into the host cells. Gu et al. identified 12 surface receptors with diverse S-binding affinities and patterns that mediate SARS-CoV-2 infection. Receptors like ASGR1 and KREMEN1 play an important role in ACE2-independent virus entry. These different host receptors may be contributing to the multiorgan tropism of SARS-CoV-2 [43].

Systemic Inflammatory Response Secondary to Cytokines Release Syndrome

A hyperactive host immune response to SARS-CoV-2 infection resulting in an excessive inflammatory reaction with a release of a large amount of pro-inflammatory cytokines, also known as “cytokine storm,” has been recognized in patients with COVID-19 [44]. SARS-CoV-2-infected cells release inflammatory mediators and chemokines that cause neutrophil aggregation. Neutrophils further secrete cytokines and chemokines that promote the accumulation of immune cells, which in turn cause immune overreaction and cytokine storms, leading to acute respiratory distress syndrome (ARDS), sepsis, and fulminant multiorgan failure [45].

In the early stage of SARS-CoV infection, there is an initial delayed release of cytokines and chemokines, followed by low antiviral interferon levels and high levels of pro-inflammatory cytokines and chemokines. Then, rapidly elevated cytokines and chemokines attract a large number of inflammatory cells, such as neutrophils and monocytes, causing tissue damage. SARS-CoV-2 is speculated to be similar and causes ACE2-expressing cells to secrete cytokines that may lead to a cytokine storm and damage multiple organs [42]. Huang et al. found that patients admitted to the ICU were found to have higher plasma levels of interleukin (IL)-2, IL-7, IL-10, granulocyte colony-stimulating factor (G-CSF), recombinant human interferon-induced protein-10 (IP-10), macrophage inflammatory proteins (MCP1, MIP1A), and TNF-α compared with non-ICU patients [46]. Cytokine storms are thought to be one of the major causes of the worsening of COVID-19, leading to ARDS and extrapulmonary multiple organ failure that may also involve the digestive system.

Histamine has also been postulated as a possible mediator of cytokine storm in COVID-19. One study found that a combination of histamine-1 and histamine-2 antagonists reduced the progression of symptom severity and improved the outcome of inpatients hospitalized for COVID-19, possibly by reducing the histamine-mediated pulmonary cytokine storm [47].

Direct Intestinal Mucosal Damage and the Role of the Intestinal Microbiota

Some studies showed that SARS-CoV-2 infection of gut epithelial cells could induce dysbiosis, intestinal inflammation, and gastrointestinal symptoms [48]. The intestinal microbiota is important for reducing COVID-19 complications. Changes in the composition and function of the gastrointestinal microbiota can affect the respiratory tract through the mucosal immune system, as the digestive and respiratory tracts are believed to influence each other through the “gut-lung axis” [49]. As an example, studies have shown that butyric acid produced by the intestinal microbiota can inhibit cytokine storms through its broad anti-inflammatory activities [50].

Wang et al. found that the lung-derived C-C chemokine receptor type 9 (CCR9) + CD4+ T-cells, which can then be recruited into the small intestine through CCL25 expressed in the small intestinal epithelium, were increased after viral infection. Once entering the small intestine, the CCR9+ CD4+ T-cells destroy the homeostasis of the intestinal flora, promoting polarization of T helper 17 cells (Th17) cells in the small intestine. Subsequent overproduction of IL-17A leads to neutrophil recruitment and causes intestinal immune damage, diarrhea, and other gastrointestinal symptoms. Through the gut-lung axis, intestinal inflammation can, in turn, affect the lung immune response and inflammation as cytokines and bacteria enter the bloodstream and reach the lung [51].

A growing number of studies that evaluated the effect of probiotic administration in reducing the incidence and severity of viral respiratory infections support its potential beneficial effects [48]. Given the potential role of probiotics in other respiratory infections, it is thought that probiotics may be useful as adjunctive therapy for COVID-19 [48]. Probiotics affect the innate and adaptive immune system and have immunomodulatory effects, inducing pro-inflammatory or anti-inflammatory immune responses to confer immunological protection to the host. It has an influence on cytokine production by intestinal epithelial cells, stimulation of IgA secretion for mucosal immunity, activation of phagocytosis and macrophage production, modulation of regulatory cells, and induction of dendritic cell maturation [52].

GI clinical manifestations and complications of COVID-19

There have been conflicting results regarding disease severity and mortality among those with and without gastrointestinal symptoms in COVID-19 patients [53-54]. Patients with gastrointestinal symptoms showed significantly higher C-reactive protein, lactate dehydrogenase, α-hydroxybutyrate dehydrogenase, alanine aminotransferase, aspartate aminotransferase, and bilirubin levels compared to those without GI symptoms [55]. According to a systematic review of 128 studies, hypoalbuminemia, abnormal GGT, and aminotransferases were more frequent in severe disease [56]. The GI clinical manifestations of COVID-19 have been summarized in Table 1.

**Table 1:** Gastrointestinal manifestations of COVID-19 COVID-19: coronavirus disease 2019

Gastrointestinal manifestations of COVID-19
Anorexia
Anosmia
Ageusia & Dysgeusia
Nausea
Vomiting
Diarrhea
Abdominal pain/discomfort
Abdominal distention
Feeding intolerance
Hematochezia
Melena
Hematemesis

Diarrhea

Among the gastrointestinal symptoms, diarrhea was the most common, followed by nausea and vomiting [57-58]. The reported incidence of diarrhea in COVID-19 varies from 2% to 49.5%. Diarrhea is thought to be due to ACE2-expressing intestinal epithelial cell invasion by SARS-CoV-2, causing local damage and subsequent diarrhea. Diarrhea can also be due to indirect damage from the inflammatory response triggered by SARS-CoV-2 infection. Medications including antibiotics could also contribute to diarrhea in COVID-19 patients.

One study that observed the GI manifestations of COVID-19 described that diarrhea started one to eight days (median time 3.3 days) since the onset of the disease with an average duration of 4.1 ± 2.5 days. Patients had an average of 3.3 ± 1.6 bowel movements per day but could have up to nine times a day [59-60]. Stool studies revealed positive fecal microscopic leukocytes in only 5.2% of cases without red blood cells, and in another study, stool cultures and fecal leukocytes were negative in all cases. These findings are consistent with viral gastroenteritis [60-61]. The intestinal bacterial profile is thought to be related to the severity of COVID-19. Patients with diarrhea are prone to dysbiosis of the intestinal flora that may further exacerbate inflammation via the gut-lung axis and result in more severe disease [62]. In line with the speculation, some studies have found that diarrhea was associated with the need for hospitalization, severe disease, ventilator use, ICU admission, and ARDS in COVID-19 patients [63-64]. 

Anorexia, Nausea, and Vomiting

Anorexia is another common gastrointestinal manifestation of COVID-19 patients, occurring in 39.9-50.2% of patients [59]. One early meta-analysis reported anorexia as the most common GI symptom (26.8%), followed by diarrhea (12.5%), and nausea/vomiting (10.2%) [5]. In another meta-analysis, nausea and vomiting occurred in 6.3% of cases, following diarrhea (11.5%), which was the most common GI symptom [57]. Among patients with severe COVID-19 presenting as ARDS requiring ICU admission, one-fifth of the patients had nausea or vomiting. Patients admitted to the ICU were also more likely to report anorexia compared to non-ICU patients [65-66].

Abdominal Pain

Abdominal pain is less frequently reported in patients with COVID-19 compared to other GI symptoms. Its incidence varies upon studies ranging from 3.6% to 6.0%. Notably, abdominal pain was observed more frequently in patients with severe COVID-19 admitted to the ICU than non-ICU patients with mild disease [60,65].

GI Bleeding

According to an analysis that reviewed 2,023 cases in 15 studies, GI bleeding occurred in 4%-13.7% of patients [60]. Massironi et al. shared the endoscopic and histologic findings of 38 COVID-19 patients [67]. The most common indication for endoscopy was GI bleeding. Esophagogastroduodenoscopy (EGD) of 24 patients and colonoscopy of 20 patients revealed that 70%-75% of cases had active lesions. The main findings on EGD included esophagitis in five cases (20.8%), bulbar ulcer in five (20.8%), erosive gastritis in four (16.6%), neoplasm in two (8.3%), and Mallory-Weiss tear in one patient (4.1%). Colonoscopy findings were significant for segmental colitis associated with diverticulosis in five cases (25%), colon ischemia in four (20%) that was confirmed histologically, diffuse hemorrhagic colitis in one, and neoplasm in one. The colonic mucosa appeared grossly normal in three patients, but histologic examination showed evidence of colitis (2 microscopic colitis, 1 lymphocytic colitis) [67]. Long-term hypoxemia with subsequent tissue hypoxia may lead to cell necrosis of gastrointestinal mucosal cells, and result in ulceration and bleeding. Treatment with corticosteroids and nonsteroidal anti-inflammatory drugs (NSAIDs), thromboprophylaxis with low molecular weight heparin, and physiologic stress secondary to severe illness could also affect the mucosa of the GI tract and contribute to bleeding [59]. Ischemic injury can also be a part of thrombotic complications of COVID-19 from excessive inflammation, platelet activation, and endothelial dysfunction. Another possibility is transient hypotensive episodes or a state of shock related to sepsis, leading to ischemic colitis [67-68].

Biliary manifestations of COVID-19

There have been reports of acute cholecystitis in COVID-19 patients, most of whom were critically ill. Most reported cases of cholecystitis associated with COVID-19 were acalculous cholecystitis, which occurs due to the hypomobility of the gall bladder, and is seen in critically ill patients with sepsis and mechanically ventilated [69-73]. Given the broad expression of ACE2 in human tissues, including in the liver, gallbladder, and bile ducts, there is a possibility of the virus playing a primary role in the development of cholecystitis via direct invasion and replication in the gall bladder and bile ducts. Detection of viral RNA in bile and gall bladder wall further supports this speculation. Other possible mechanism includes inflammation from cytokine release syndrome, ischemia from hypercoagulability, and thrombotic microangiopathy secondary to SARS-CoV-2 infection [69,71-72,74].

Hepatic manifestations of COVID-19

**Figure 3:**
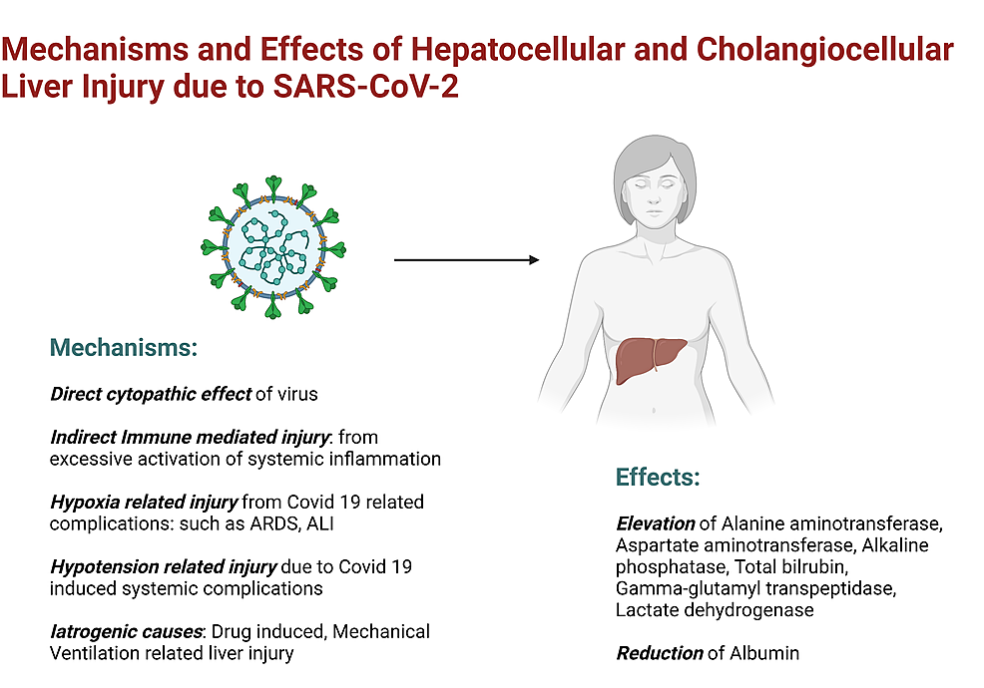
Mechanism of liver injury in COVID-19 patients SARS-CoV-2: severe acute respiratory syndrome coronavirus 2; COVID-19: coronavirus disease 2019; ARDS: acute respiratory distress syndrome; ALI: acute liver injury

Abnormal liver function reflecting hepatocellular and cholangiocellular injury is often encountered in patients with COVID-19 with a pooled prevalence of 24.4% during the course of the disease [75] and was seen in up to 76.3% of hospitalized patients in one study [76]. Abnormalities are often manifested as elevated alanine aminotransferase (ALT), aspartate aminotransferase (AST), alkaline phosphatase (ALP), total bilirubin, gamma-glutamyl transpeptidase (GGT), lactate dehydrogenase (LDH) levels, and decreased albumin levels. A recent meta-analysis showed a pooled incidence of elevated AST/ALT of about 20%, hyperbilirubinemia of 13.4%. Although the cholestatic pattern of elevated liver enzymes was rarely reported initially, recent reviews show that elevation of ALP and GGT were seen in 6.1% and 21.1% of patients, respectively. The pooled incidence of hypoalbuminemia was 55.5% while patients with severe disease had significant hypoalbuminemia reaching up to 72.9% [75]. Importantly, several studies suggest that COVID-19 patients with transaminitis were at higher risk for severe disease and increased mortality [77-79]. Liver injury may be a direct effect of the virus, a hepatic manifestation of multiorgan failure in a complicated disease course, decompensation of underlying liver disease, or iatrogenic [80]. The mechanisms of liver injury in COVID-19 patients are likely multifactorial and include the direct effect of the virus, indirect immune-mediated injury from excessive systemic inflammation, ischemic injury from hypoxia or hypoperfusion secondary to COVID-19-related complications, and iatrogenic causes (drug-induced, mechanical ventilation) [80-81]. The probable mechanisms of liver injury in COVID-19 have been presented in Figure 3.

Direct Cytopathic Effect of the SARS-CoV-2 Virus

Some evidence to suggest direct viral injury to the liver by SARS-CoV-2 has been found in postmortem liver biopsies of two COVID-19 patients with elevated aminotransferases. Ultrastructural examination with transmission electron microscopy identified coronavirus particles in the cytoplasm of hepatocytes. Histological examination of SARS-CoV-2 infected hepatocytes showed mitochondrial swelling, endoplasmic reticulum dilatation, and decrease in glycogen granules along with massive hepatocyte apoptosis and binuclear hepatocytes, indicative of direct cytopathy caused by SARS-CoV-2 [82]. However, there have also been some skeptical views, arguing that such findings could be confused with other normal physiologic structures and changes in postmortem tissue. In addition, hepatocytes express little or no ACE2 receptors, which acts as a cellular entry point for SARS-CoV-2, making direct viral entry to hepatocytes less likely [6,32]. However, some studies found that hepatic ACE2 expression increases in inflammatory liver conditions and in fibrotic/cirrhotic liver [81,83]. This could explain other studies showing worse COVID-19 outcomes in those with chronic liver disease [84]. Hepatocellular ACE2 expression is also affected by hypoxia [83]. Organotropism of SARS-CoV-2 beyond the respiratory tract, including the liver, has been demonstrated by the detection of viral RNA through reverse transcription-polymerase chain reaction (RT-PCR). Such discoveries suggest that direct cytotoxicity from active viral replication of SARS-CoV-2 in the liver is a possible mechanism for liver injury in COVID-19 patients [85]. According to a study, histological findings in patients infected with COVID-19 showed alterations of the intrahepatic blood vessel network, such as increased number of portal vein branches with massive lumen dilatation, partial or complete luminal thrombosis of the portal and sinusoidal vessels, fibrosis of portal tract, focally markedly enlarged and fibrotic. Such findings suggest that life is not the main target of significant inflammatory damage but rather reflects secondary changes caused by systemic response induced by the virus [86].

Viral entry into the circulation through damaged alveolar epithelial cells and subsequently into the liver that has abundant dual blood supply can be another possible route of hepatic dissemination. Additionally, retrograde infection of the liver from SARS-CoV-2 infection of the intestinal tract through the portal vein is also possible. The “gut-liver axis” refers to the bi-directional relationship between the intestine, its microbiota, and the liver through the portal vein, biliary tract, and systemic circulation [87]. Host and intestinal metabolites transferred from the intestine to the liver through the portal vein can affect liver function when it binds to the toll-like receptors of hepatocytes and cause inflammation. This may explain the abnormal liver function test results commonly encountered in COVID-19 patients. On the other hand, the liver releases bile acids and bioactive media into the intestine via the biliary tract and systemic circulation [88].

These hypotheses can be backed by the in situ hybridization analyses revealing SARS‐CoV‐2 virions in the vessel lumens and endothelial cells of portal veins of COVID‐19 liver specimens [86], and the fact that patients with intestinal injury were more likely to have a severe liver injury.

Drug-Induced Liver Injury (DILI)

Being the major organ for drug metabolism, liver injury can be caused by medications used in the treatment of COVID-19. The drug metabolites can cause cellular stress that can lead to apoptosis or necrosis of liver cells. Ritonavir was associated with transaminitis in 44% of COVID-19 patients in one study, and Zhan et al. found lopinavir/ritonavir to be an independent risk factor for severe liver injury in COVID-19 patients [89-90]. Other medications, such as hydroxychloroquine, azithromycin, tocilizumab, remdesivir, and glucocorticoids, have also been linked to elevated serum transaminase [80].

Immune Dysregulation

Some patients can develop an excessive systemic inflammatory response to SARS-CoV-2 infection, leading to the rapid production of a large number of pro-inflammatory cytokines such as interferon-α, interferon-γ (IFN-γ), interleukin-1β (IL-1β), interleukin-2 (IL-2), interleukin-6 (IL-6), interleukin-8 (IL-8), interleukin-10 (IL-10), and TNF-α [91]. The liver can also be affected by the indirect immune-mediated inflammatory injury caused by the cytokine storm. Supporting this speculation, studies have found that increased IL-6 and IL-10, and decreased CD4+ T cells were independent risk factors for severe liver injury.

Ischemic Injury From Shock/Hypoperfusion

Hypoxic injury of the liver can occur in patients with COVID-19. Causes for hypoxia can be multifactorial, secondary to many conditions that can co-exist with severe COVID-19, including hypoxemia due to hypoxic respiratory failure/ARDS, right ventricular dysfunction and subsequent hepatic congestion from pulmonary thrombotic complications or mechanical ventilation, organ hypoperfusion/microcirculation disruption due to hypercoagulable state, diffuse endothelitis, or shock. Hypoxia-induced upregulation of ACE2 expression in hepatocytes may also further worsen liver injury by SARS-CoV-2 [92-93].

Pancreatic manifestation of COVID-19

The pancreatic involvement of COVID-19 ranges from an asymptomatic mild elevation of serum amylase and/or lipase levels to acute pancreatitis and some with further progression to necrotic pancreatitis [94-95]. Though it is one of the criteria for the diagnosis of acute pancreatitis, an elevated lipase level has been observed in nearly one-third of COVID-19 patients with ARDS without other clinical or imaging evidence of pancreatitis. This is thought to be due to impaired microcirculation with subclinical cellular damage in severely ill patients. Thus, pancreatic enzyme elevation should be interpreted with caution along with clinical and/or image findings of pancreatitis since other factors, such as gastroenteritis, renal failure, hypoperfusion related to critical illness, and medications, can all affect the pancreatic enzyme levels in the absence of clinically significant pancreatitis [96-97]. One retrospective observational cohort study that reviewed 48,012 hospitalized patients reported a point prevalence of pancreatitis among COVID-19 patients to be 0.27%. The study also revealed idiopathic pancreatitis was the most common etiology (69%) among those with COVID-19, in contrast to gallstone and alcohol being the most common causes for pancreatitis in the general population without COVID-19 [98]. This leads to plausible speculation that SARS-CoV-2, like a few other viruses known to cause pancreatitis, may directly cause injury to the pancreas through its highly expressed ACE2. Detection of SARS-CoV-2 from pancreatic pseudocyst fluid adds support to this theory. Alternatively, indirect injury from a systemic inflammatory response to SARS-CoV-2 infection or hypoperfusion and ischemic injury from diffuse severe endotheliitis and/or the thrombogenic state related to COVID-19 can also explain the pancreatic damage [99-100]

In addition, virus-mediated lipotoxicity is another possible mechanism of pancreatic injury. As the virus targets the pancreas, interstitial leakage of pancreatic lipase may occur and result in adipose lipolysis, leading to increased levels of unsaturated fatty acids. The toxic fatty acids then cause mitochondrial injury and stimulate a cytokine storm that can lead to disease progression with multiorgan failure and possibly even death. The adipocytes also express ACE2 and maybe another direct target of the virus and contribute to the production of toxic unsaturated fatty acids through adipose triglyceride lipase [101-102]. However, the exact mechanism remains unclear and further studies are needed to establish a causal relationship between SARS-CoV-2 and pancreatitis.

Complications include pancreatic necrosis, peripancreatic fluid collection, walled-off necrosis, pseudocyst formation. New-onset diabetes has also been reported as sequelae of acute pancreatitis in COVID-19 patients, though the possibility of COVID-19 infection being a mere coincidence cannot be ruled out [103]. Studies showed that patients with acute pancreatitis and concomitant COVID-19 were at increased risk for severe disease requiring mechanical ventilation and/or ICU admission, persistent organ failure, and worse clinical outcomes, including longer hospital stay and mortality compared to those without co-existing pancreatitis [98,104].

Future prospects

Although further studies should elucidate the possibility of fecal-oral transmission of SARS-CoV-2, the importance of good hand hygiene cannot be emphasized enough in addition to standard protection including facemasks and social distancing. Once the fecal-oral transmission route is confirmed, implementation of stool PCR to test for clearance with an extended period of isolation strategy to minimize disease transmission may be helpful in confining disease spread. It is important to understand that COVID-19 patients can present with GI symptoms and should prompt clinicians to consider it as a differential diagnosis in a proper clinical context. Recognition of risk factors, including those related to GI conditions, and prioritizing patients at risk for disease progression is important for early intervention and minimizing disease severity. More understanding of the involvement of the microbiota and the gut-lung axis and its immunoregulatory role in COVID-19 is needed, and future studies on the possible benefit of probiotics as an adjunctive treatment are of interest.

## Conclusions

Patients with COVID-19 can present with a variety of GI symptoms, such as diarrhea, anorexia, nausea, and vomiting, and can be associated with several conditions, including liver injury, GI bleeding, acute cholecystitis, and acute pancreatitis. It is essential to understand that COVID-19 patients can present with GI symptoms, and clinicians should consider it a differential diagnosis in the proper clinical context. Detection of SARS-CoV-2 RNA in fecal samples could be possibly used as a potential marker for confirming viral clearance in the future. With growing knowledge of the immunological importance of the intestinal microbiota and the gut-lung axis, its association and role in regulating the disease severity of COVID-19 are of interest. Probiotics may have beneficial effects in attenuating disease severity, and further studies are needed to investigate its potential as an adjunctive therapy.
